# Quality of life beyond measure: Advanced cancer patients, wellbeing and medicinal cannabis

**DOI:** 10.1111/1467-9566.13684

**Published:** 2023-06-07

**Authors:** Alexandra Smith, Rebecca E. Olson, Nathalia Cordeiro da Costa, Maddison Cuerton, Janet Hardy, Philip Good

**Affiliations:** ^1^ School of Social Science The University of Queensland Brisbane Queensland Australia; ^2^ School of Health and Rehabilitation Sciences The University of Queensland Brisbane Queensland Australia; ^3^ School of Public Health The University of Sydney Sydney New South Wales Australia; ^4^ Department of Palliative and Supportive Care Mater Health Services Mater Research‐University of Queensland South Brisbane Queensland Australia; ^5^ Department of Palliative Care St. Vincent's Private Hospital Brisbane Brisbane Queensland Australia

**Keywords:** Australia, cancer, Deleuze and Guattari, medicinal cannabis, palliative care, wellbeing

## Abstract

Experiences of advanced cancer are assembled and (re)positioned with reference to illness, symptoms and maintaining ‘wellbeing’. Medical cannabis is situated at a borderline in this and the broader social domain: between stigmatised and normalised; recreational and pharmaceutical; between perception, experience, discourse and scientific proof of benefit. Yet, in the hyper‐medicalised context of randomised clinical trials (RCTs), cancer, wellbeing and medical cannabis are narrowly assessed using individualistic numerical scores. This article attends to patients’ perceptions and experiences at this borderline, presenting novel findings from a sociological sub‐study embedded within RCTs focused on the use of medical cannabis for symptom relief in advanced cancer. Through a Deleuzo–Guattarian‐informed framework, we highlight the fragmentation and reassembling of bodies and propose body‐situated experiences of wellbeing in the realm of advanced cancer. Problematising ‘biopsychosocial’ approaches that centre an individualised disconnected patient body in understandings of wellbeing, experiences of cancer and potential treatments, our findings foreground relational affect and embodied experience, and the role of desire in understanding what wellbeing is and can be. This also underpins and enables exploration of the affective reassembling ascribed to medical cannabis, with particular focus on how it is positioned within RCTs.

## INTRODUCTION

Cancer disrupts wellness. Diagnosis, disease and associated symptoms can unsettle a person’s physical, psychological and social functioning (Ranchor et al., 2010; Reeve et al., 2010). Commonly used clinical measures of ‘quality of life’ reflect such losses, drawing on tools that translate wellbeing into biopsychosocial functioning (Hui & Bruera, 2016). Medical cannabis (MC) is one option for alleviating effects of cancer and anti‐cancer treatments, potentially disrupting symptoms and the experience of disease. Situated bodies that are often deemed problematic intersect the disruptive experiences of both cancer and cannabis.

A Deleuzo–Guattarian account of bodies and assemblages, however, has the potential to (re)focus our attention on *what bodies can do*. Shifting beyond a compartmentalised body/mind/society approach, it fosters an appreciation of embodied processes and flux, the movement and dis‐connection of bodies in social spaces and the operation of desire. In this paper, we work from such a framing, drawing on the writings of Grosz (1995), Sointu (2013, 2006) and Coffey (2022), to critically explore experiences of bodies‐with‐cancer and accompanying articulations of wellbeing. From this positioning, wellbeing is considered in the context of the clinic, and the social, experiential body. Together, this lays a foundation for our critical exploration of findings from a sociological sub‐study, embedded in a series of randomised clinical trials (RCTs) of MC for the treatment of advanced cancer symptoms, conducted in one Australian city. This exploration reimagines the wellbeing‐MC nexus, focusing on those who are affected by advanced cancer and its associated symptoms, including pain, and enabling examination of wellbeing and the situated use of MC.

### Wellbeing in the clinic: Cancer, pain and measuring quality of life

Cancer disrupts bodies and unsettles wellbeing. For those living with cancer, pain is the most prevalent symptom at diagnosis (Breivik et al., 2009), with prevalence increasing over the disease trajectory (van den Beuken‐van Everdingen et al., 2007). Cancer pain can affect all aspects of a person’s life (Haumann et al., 2017)—generating disruptive thoughts (Brenne et al., 2013), impacting relationships (Rabin, 2019), limiting everyday activities (Russo & Sundaramurthi, 2019) and often precipitating or accompanied by other physical symptoms: insomnia, fatigue and significant mobility limitations (Chien et al., 2021; Savard & Morin, 2001). Pain’s presence is often perceived as a reminder of the cancer (Liu et al., 2018), hampering future‐focused thoughts and undermining expectations of recovery (Torresan et al., 2015). People who experience cancer pain cast it as a threat to their social identity and certainty about the future (Liu et al., 2018; Rafii et al., 2021), compounding experiences of biographical fracture (Reeve et al., 2010) and the threats to personhood posed by cancer and associated interventions (Hubbard et al., 2010). Such pain is often accompanied by feelings of helplessness (Okano et al., 2001), fear (Gibbins et al., 2013), anger (Torresan et al., 2015), depression (Aryankhesal et al., 2019) and anxiety (Walker et al., 2021); those with higher pain levels report mood disturbances and negative emotions (Sutton et al., 2002). Pain itself is also affected by psychological factors: Those with a history of distress and anxiety are more likely to experience cancer pain (Moloney et al., 2021). Likewise, social factors shape cancer pain: Those who are employed, with higher incomes and access to health care, report lesser overall cancer pain than their respective counterparts (Ham et al., 2017). Contributing to this complexity, cancer pain tends to be undertreated (Adam & Murchie, 2014), with negative consequences for patients’ quality of life, emotional and mental health and ability to engage in daily physical and social activities (Dabbous et al., 2017). Cancer pain is thus a predominant, complex and multidimensional experience interwoven with psychological, social and disease‐related factors.

Because cancer pain involves multiple aspects which together influence its perception and experience (Ahles et al., 1983), a biopsychosocial model (Engel, [1977] 2012)—considering physical, social, psychological and behavioural dimensions of illness—has long been advocated in assessing and managing cancer pain (King, 2016; Syrjala & Chapko, 1995). Yet, the model’s application in clinical practice and research—using numerical rating scales such as the Edmonton Symptom Assessment Scale (ESAS) to appraise pain (Hui & Bruera, 2016)—remains fraught. Not only does the biopsychosocial model compartmentalise intersecting bio‐psycho‐social features into discrete categories, in application, physiological aspects continue to dominate (Mescouto et al., 2022). Furthermore, the practice of numerically measuring pain can be problematic: Patients may not equate ‘pain control’ with being pain‐free or attaining a specific score, instead considering their ability to perform functions as important in assessing whether pain is under ‘control’ (Bhatia et al., 2014; Gibbins et al., 2013). Both patients and caregivers suggest that reducing the impact of pain on emotional wellbeing and daily life is more important than alleviation (Luckett et al., 2013). Thus, there is critique of using numerical rating scales as tools in trials for assessing cancer pain interventions, such as cannabinoids (Boland et al., 2020).

### Wellbeing on trial: Medical cannabis and cancer

In the milieu comprised of cancer, pain and measures of wellbeing, cannabis occupies conceptually complex and shifting positions. The legalisation of cannabis for use in health‐related settings in various countries has been accompanied by increased interest in its pharmacology, clinical efficacy, regulation and sociocultural, structural and experiential elements involved in its use and effects (e.g. Gardiner et al., 2019; Kim et al., 2019; Kosiba et al., 2019; Lintzeris et al., 2020; Luckett et al., 2016; Martin & Bonomo, 2016; Panozzo et al., 2020; Rapin et al., 2021). In Australia, cannabinoids are legally available for medical use through varied state‐based regulatory schemes, with prescription required from an authorised health professional (Graham et al., 2022; Martin et al., 2020). Several clinical drug trials and other studies have been implemented to investigate MC, involving a blending of RCT and ‘Real World Evidence’ approaches (Graham et al., 2020), with one focus being cannabinoids in symptom relief for advanced cancer patients in palliative care (Good et al., 2019; Martin et al., 2020).

Complementing and at times contrasting this clinical realm, social science perspectives have been brought to bear, drawing from long‐standing interests in drug use, medicalisation and stigma (Athey et al., 2017; Becker, 1953; Järvinen & Ravn, 2014; Morris, 2020). Through such lenses, MC is comprehended as a contested substance and space: multiple, often conflicting, meanings are assigned to it; perceptions of its benefits and risks are wide‐ranging; and intensive work is undertaken at the often‐blurred boundary between licit and illicit and medical and recreational use (Kvamme et al., 2021; Pedersen & Sandberg, 2013; Zarhin et al., 2018). In Norway, where access to MC is not legislatively supported, Pedersen and Sandberg (2013) interrogate cannabis users’ conceptualisations of cannabis as therapeutic and their illegal use of it as ‘medicalised revolt’. In Israel, by contrast, legal MC access is well‐established. Here, Zarhin et al.’s (2018) work highlights the challenges operating at the intersection of ‘public opinion’, cannabis users’ experiential perspectives and the positions of medical professionals, clinical researchers and policymakers.

### Wellbeing in the body: Assembling, connecting and relational affect

Against this clinical background, analysis of ‘wellbeing’ features within social science research on life‐affecting and life‐limiting illness, including cancer. Nyvang et al.’s (2016) work conceptualises ‘wellbeing’ beyond a specific bodily health issue to a more encompassing whole‐of‐life e/affect. In studies with cancer patients, individuals describe disruption to their emotional and embodied selves (Hubbard et al., 2010) and a shift into a ‘low‐control’ domain (Ranchor et al., 2010). Body‐, emotion‐ and identity work are used to re‐imagine the self and sense of how ‘wellbeing’ might be experienced following a cancer diagnosis (Reeve et al., 2010; Sulik, 2009). Wellbeing is thus positioned within a personal, embodied and experiential realm.

Recent work extends on this, foregrounding a shift in conceptualisations of wellbeing, including its positioning relative to disease. Sointu (2016, p. 312) presents an alternative reading of wellbeing drawn from a ‘more‐than‐human’ approach to embodiment and wellness in complementary and alternative medicine, premised on the ‘intertwining of affective experience and discursive meaning’. This reading considers the intersubjective operation of control and agency as ‘created through complex connections between social ideals, constructions of the body, and the performance of embodied health practices’ (Sointu, 2006, p. 204). Coffey (2022)—extending on Sointu’s (2006, 2013, 2016) and McLeod’s (2017) work, which draws on a novel Deleuzo–Guattarian approach to the sociological examination of health and wellbeing—analyses the assembling of youth wellbeing: the body, conceptualisations and experiences of wellbeing are seen as socially inscribed, structurally mediated and discursively shaped while also comprising embodied practices and relational affect. Together, these give shape to complex assemblages: A multiplicity of dis‐connections and reconnections between fragmented and reassembled bodies. The body is here (re)instated as central to wellbeing: ‘bodies and affective relations… [are] taken as crucial participants in producing selves and socialities’, with wellbeing ‘assembled through the dynamics of everyday life’ (Coffey, 2022, p. 70).

A Deleuzo–Guattarian account of bodies importantly focuses attention on *what bodies can do*, explicitly shifting beyond dualist mind/body—or segmented bio/psycho/social—approaches. Instead, there is appreciation of process, flux and the movement—or de‐reterritorialisation—of bodies within cartographies of social space. In this space, bodies are appreciated through their a/effects and sometimes‐unpredictable interconnections with other bodies, rather than final static positions or functions (Deleuze & Guattari, 1987). Social formations, or assemblages, may be *machinic*—composed of physical objects and ‘actions and passions, an intermingling of bodies reacting to one another’ (Deleuze & Guattari, 1987, p. 103). They may also be *collective assemblages of enunciation*, composed of ‘acts and statements, of incorporeal transformations attributed to bodies’ (Deleuze & Guattari, 1987, pp. 102–103). This rhizomatic approach to understanding the body‐in‐movement and bodies‐in‐connection helps to examine body practices, assemblage re‐formation and modes of thinking as positioned and always relational (Deleuze & Guattari, 1983).

Such assemblages are understood as held together by ‘desire’—a ‘positive productive force’ that is ‘not just a stabilising but also a destabilising force’ (Muller & Schurr, 2016, p. 8); the assemblage ‘does not exist without the passions the assemblage brings into play [or]…without the desires that constitute it as much as it constitutes them’ (Deleuze & Guattari, 1987, p. 65). Desire, then, enables an alternate and analytically powerful reading of bodies. In this paper, we attend to interconnection and movement of bodies–desires–socialities as assemblages. This enables examination of the forces driving particular body practices: MC in relation to advanced cancer.

As noted previously, the experiential, discursively shaped and embodied nature of wellbeing can be challenging to comprehend in clinical contexts. This is so regarding cancer, where physical *un*wellness manifests in symptoms such as pain, which are then treated with discursively loaded approaches such as MC. Acknowledging these challenges, as well as those relating to clinical trial design, implementation and recruitment in palliative care, advanced cancer and MC spaces (Good et al., 2019), a qualitative sub‐study was embedded as part of a series of larger, multicentre trials of MC in advanced cancer, conducted in one Australian metropolitan area with palliative care patients. Trial designs explicitly prioritised assessing overall symptom burden and general improvement in wellbeing as indicators of patients’ responses to MC (Good et al., 2020; Hardy et al., 2020, 2022); however, as noted earlier, numerical rating scales pose limitations. As such, this qualitative sub‐study was designed to elicit in‐depth understanding of patients’ expectations, experiences and perceptions of their own health and cannabis use (prior, current, and trial‐related); inform future trials design; and explore patients’^1^ conceptualisations of wellbeing and quality of life in the context of terminal illness. The results and analysis presented here extend on findings arising from this embedded research (Olson, Smith, Good, et al., 2022, Olson, Smith, Huggett, et al., 2022), drawing on the Deleuzo–Guattarian frame outlined above. We situate this analysis and discussion of its implications at a little‐studied intersection comprised of embodiment, affect and sociocultural discourse in patient experiences; and of cancer, clinical trials and medical cannabis. In doing so, we offer sociological insight, supporting the value of embedded qualitative studies in the study of patient experiences of cancer, palliative care and the emergent domain of clinical trials of medical cannabis.

## METHODS

A qualitative sub‐study—using semi‐structured interviews—was embedded within three MC trials conducted by the broader research team (Good et al., 2020; Hardy et al., 2020, 2022). Using purposive sampling techniques in 2019–2020, we identified potential interviewees from two palliative care patient cohorts: those who were eligible but declined participation in one of three consecutively‐run MC RCTs; and patients who were eligible and consented to trial participation and who were subsequently randomised to a placebo or intervention (THC and/or CBD investigational product) arm, with continued use of existing medications. Patients potentially meeting inclusion criteria for the MC trials were approached by clinicians and referred to a nurse overseeing recruitment; following written consent, an interview was scheduled at either a hospital (*n* = 42), or by telephone (*n* = 6), at times suitable for patients and their carers where applicable. An experienced qualitative researcher oversaw interview data collection and remained part of the investigative team for the duration of the trials. This approach supported the collection of rich findings into patients’ discursively shaped, and embodied experiences of advanced cancer, recreational cannabis and MC. Of note, in discussing participants’ perceptions, experiences and expectations, the vast majority described past or current use of cannabis.

A total of 48 patients (28 trial participants; 20 non‐participants) were interviewed. Interviews took place before or during trial participation (where applicable), lasted 20–60 min and were facilitated by two experienced interviewers with backgrounds in sociology and social work. Guiding interview questions prompted patients to reflect on their perspectives of MC and general marijuana use; reasons for participating or not in a trial; and awareness of and views on changing MC laws. Each interview was recorded using a digital voice recorder and transcribed verbatim. Socio‐demographic details—also reported elsewhere (Olson, Smith, Good, et al., 2022; Olson, Smith, Huggett, et al., 2022)—reflected a balance of characteristics: 47.91% (*n* = 23) male, 52.08% (*n* = 25) female; 81.25% living with a spouse or children, 14.58% (*n* = 7) living alone; and most common cancer diagnoses including breast (25% (*n* = 12)), prostate (20.83%, *n* = 10) and lung (14.58%, *n* = 7).

Our approach to analysis was ‘hybrid’, valuing the generative capacity of conventional qualitative methods, whilst seeking to engage the ‘multiverse of opportunities to think with… theories’ (Brown et al., 2021, p. 232). First, we drew on well‐established techniques, analysing transcripts thematically and by case (Braun & Clarke, 2019). Through open coding—undertaken by two research team members with advanced knowledge in the health social sciences—we identified patterns and compared cross‐data sources. Nvivo 12 software facilitated data management and code organisation, resulting in seven core themes, with a further 6–10 sub‐themes each. This paper focuses on one core theme: wellbeing (for other findings, see Olson, Smith, Good, et al., 2022; Olson, Smith, Huggett, et al., 2022).

Guided by critiques of the rigidity of traditional qualitative methods as ‘methodological fetishism’ (Olson & Dadich, 2022), and calls to foreground theory, in the latter stages of analysis, we supplemented thematic analysis with post‐qualitative practices of thinking with theory (Mazzei, 2021). We drew on Sointu (2006) and Coffey’s (2022) Deleuzo–Guattarian theorisations to counter the segmented understandings of wellbeing employed in standardised RCT measurements and foreground appreciation of wellbeing as embodied, relational, socially inscribed, structurally mediated and discursively shaped. Specifically, we brought ‘concept and problem together’ (Mazzei, 2021, p. 198) to achieve our aim of reconsidering what wellbeing is and does in the context of advanced cancer and MC.

Approval for the qualitative sub‐study was secured separately from RCTs via relevant hospital ethics committees (HREC/17/MHS/97; HREC 17/27). Patients were provided with information sheets explaining the study’s aims and interview methods and informed that they were free to withdraw at any time without consequences (none chose to do so). Patients were also informed that any information provided would not affect their eligibility for the clinical trial itself nor change the care provided to them. Both those participating in a clinical trial, and those who declined, continued to receive their other medications and treatments.

## FINDINGS

Findings illuminate shifting, interconnected perceptions of illness, wellbeing and coping and expectations and embodied experiences of cancer symptoms. Interviewees described bodies, wellbeing, and assemblages disrupted by pain which precipitated loss: of functional independence, emotional and psychological stability and relational and social *inter*dependence. Themes of disassembling and loss foregrounded expectations and experiences of MC. Interviewees considered MC through intersecting lenses: medicalised understandings of physical pain, psychological difficulties and health‐related quality of life; highly personal, situated conceptualisations of self, embodiment, relational and emotional displacement; and more encompassing notions of wellbeing that elude clinical discourse. As wellbeing is the context in which medicinal cannabis is considered, the findings chart a trajectory from interviewees’ conceptualisations of wellbeing and of loss‐disassembling through expectations of the reassembling effects of MC to embodied, experiential, socially situated experiences of RCTs and MC—both on and off the trial—in particular. In the discussion, we extend on these interpretations using Deleuze and Guattari to challenge dominant conceptualisations of wellbeing in advanced cancer contexts.

### ‘I’ll take anything for the pain to go away’: Pain and embodied wellbeing

[C]ancer starts to change your life, it affects all of the things that you had and did, and they slowly get taken away…. So, your quality of life diminishes…quite rapidly… For me it’s the ability to function as an individual, without help, both physically and mentally, and just do the day‐to‐days—the very simple things that we take for granted. Once you’ve lost those and you can’t get them back… psychologically, that’s pretty hard.

(P01/18, M‐61, MC1‐CBD)^2^

Loss and disruption—attributed to experiences or expectations of cancer pain—featured in interviews. Changes, as described above by P01/18, undermined physical functioning and entangled with emotions, self‐identity, mental illness and relations. The indivisibility of the physical‐psychological‐relational experience of pain is evident in the quotes presented below, separated here for structural clarity into three sub‐sections.

#### Losing independence: Physical pain and wellbeing

Complex health concerns related to advanced cancer were often glossed as ‘pain’; quality of life was positioned as normality, free from ‘pain’ and associated limitations:

Quality of life means I’m doing everything normal: drive…cook…chores without being exhausted or feeling unwell… attend our daughter’s activities… get out and about… Not be confined to the house… I hardly ever do anything. I wake up and go and sit on the lounge. I have something to take my tablets. I have a sleep… I’m in pain so I can’t do much.

(P01/26, F‐52, MC2‐Blinded)

P01/22 (M‐71, MC1‐Placebo) similarly talked about the quality of life being an ability to ‘just get out and do things’. While describing the challenges of treatment side effects, P01/21 (F‐38, MC1‐CBD) stated her focus: ‘being able to enjoy life without being in a huge amount of pain’. Others acknowledged that their experience of symptoms and (hoped‐for) wellbeing varied day to day, with uncertainty around the cause and trajectory of symptoms:

I don’t know if I feel bad because of the chemo or the disease… Some days you just wish you could go to sleep and not wake up. Anything that makes you get away from that feeling. Hope’s the whole thing isn’t it? … Nobody expects to go out this way really. I was hoping I’d be shot by a jealous husband when I was 90, but that’s not going to happen.

(P01/12, M‐67, MC1‐Placebo)

The disassembling effect of physical pain is situated here within patients’ social domains and their desire to undertake valued, everyday activities. Pain is counterbalanced by the anticipated reassembling effect of hope: A desire for relief and a return to normalcy.

#### Losing purpose: Psychological and emotional pain

Patients reflected on pain and lacking wellbeing, referring to a further sense of loss, whereby physical limitations undermined emotional health and self‐image:

I felt that I was a useless bit of stuff around the corner. I might as well not be here. That’s the effect you get. Why be here and do that? I might as well not be here.

(P01/09, M‐77, MC1‐Placebo)

Loss of physical strength and shifting self‐image was reflected in this repositioning, underscoring the disassembling effect of cancer and related symptoms:

I got a bit depressed because I spent [a few] years building myself up. I had no fat on me at all, I was a very solid man. [Then] people didn’t even recognise me because I’d lost so much weight… my face was all drawn… I was only tiny… I liked being strong and I felt like I was weak.

(P01/17, M‐58, MC1‐CBD)

Coping with depression and anxiety, and comprehending links to physical pain, also featured in interviews:

Easier would be, I’ve got more energy. I live longer, a little bit longer… out of the pain, go and do things with my kids, don’t get tired. Have fun. Don’t sit there and sulk and make pity on yourself…. try and numb all that stuff so you can have fun.

(P01/20, F‐55, MC1‐CBD)

Sometimes I get depressed because you think too much… you think oh why me, but most of the time I seem to be coping fairly well… I’ll do anything to beat it… I’ll take anything for the pain to go away.

(P01/07, M‐67, MC1‐Placebo)

Comparatively, P01/23 (F‐70, MC1‐Placebo) described quality of life as a ‘better outlook [with] more positive attitude and feeling… If you’ve got terminal cancer, it’s very easy to get depressed… Sometimes I feel like I’ve got the world on my shoulders’. P01/17 noted the intersecting experiences of anxiety and physical pain:

I do have a bit of anxiety, so that’s one of the main reasons I changed my mind and said to [the doctor] I’ll do it, because you get a bit of anxiety, more about other people than myself… my wife was saying, you’re having anxiety, you need to do something.

(P01/17, M‐58, MC1‐CBD)

Interviewees highlighted the interplay of embodied and relational affects of physical pain, drawing together emotional impacts, physical limitations and the disassembling effects of cancer on bodies, identities, relations and mood. By situating bodies relative to time, space and affect and with reference to hope and anticipation of future relief—both physical and psychological/emotional—interviews indicated a disassembling and repositioning of the body‐in‐flux relative to cancer symptoms and wellbeing.

#### Losing in(ter)dependence: Assemblages, relational wellbeing and reclaiming normalcy

In contemplating the quality of life, interviews emphasised the ability to regain relational wellbeing and purpose:

[Quality of life means] being able to independently access my children’s wellbeing, independently accessing the community, my home. Being able to advocate for myself…. To talk for myself and be able to be of the right mind in order to do those.… [B]eing able to interact with my peers, my family, my husband, children, in a way that would not put pressure or add an extra burden to them.

(P01/25, F‐37, MC2‐Blinded)

Physical independence and wellbeing were at once interconnected—*interdependent*—with her family’s wellbeing.

Frequently, interviewees expressed desires to be able to perform physical tasks as a means of fulfiling valued functional‐relational‐emotional roles:

Staying in bed all day… or just not having the energy to walk from the bedroom to the kitchen. I tried to iron a shirt the other day… I ironed one sleeve and then I had to sit down for 15 minutes… I know that doesn’t sound much but to me I’m a mum, I’m a wife, it’s everything.

(P01/14, F‐49, MC1‐CBD)

When I’m having a good day, I’m a lot better company to be around. Plus, you’re self‐sufficient. You don’t need to have little things done for you. Whereas if you’re having a bad day… you haven’t got enough energy to get up and get yourself a little bit of water. So… I think it makes a big difference for those around you.

(P03/01, M‐57, MC1‐Placebo)

Deleuzo–Guattarian theory helps us see patients’ conceptualisations and experiences of pain and wellbeing as assemblages: Both *machinic*—composed of physical objects and of ‘actions and passions, an intermingling of bodies reacting to one another’ (Deleuze & Guattari, 1987, p. 103); and *collective assemblages of enunciation*, composed of ‘acts and statements, of incorporeal transformations attributed to bodies’ (Deleuze & Guattari, 1987, pp. 102–103). Bringing together such assemblage thinking with Sointu’s (2013) work on embodied wellbeing, and the foregrounding of relationality evident in feminist ethics of care literature (Langford, 2019), highlights the complexity and continual repositioning of bodies‐with‐cancer.

### ‘You can just participate in life’: Desiring‐bodies, relational affect, and expectations of medical cannabis

Guided by facilitators, interviewees moved from reflecting on conceptualisations of illness, wellbeing and the uncertainty of cancer symptoms, to positioning these aspects as potentially subject to the therapeutic effects of MC. In doing so, patients shifted back and forth between reflecting on multi‐dimensional aspects of quality of life, coping and normality, and a more specific focus on the physical pain they anticipated would be relieved through MC. P01/10 (F‐69, MC1‐CBD), for example, hoped MC could help her to ‘feel normal’ and be ‘pain‐free’:

I hope it can help me, my cancer. Or at least how I deal with it… Just to have days where you …feel normal and great and you can…participate in life… Quality of life is not just existing and being sick all the time and waiting to die, but actually living life until that happens, and that’s pain‐free.

(P01/10, F‐69, MC1‐CBD)

P01/23 (F‐70, MC1‐Placebo) cast her pain as emotional, discussing the depression she experienced alongside her cancer diagnosis and treatment and contemplated the potential for MC to help her ‘to maybe not be depressed and …maybe mak[e] it a little bit easier for me to bear’. Other interviewees similarly touched on the perceived potential for MC to improve their state of mind, by controlling physical symptoms:

So [others] said that, once they’ve taken it [MC]… they feel calmer. They know it’s not going to heal them but… if they’ve got the shakes or …shortness of breath, or they’re nauseated, they’ve got a headache, then it helps them. So, I thought oh well those are the things I experience, I’d like to give it a go.

(P01/25, F‐37, MC2‐Blinded)

I haven’t let the cancer take control of me. I’m taking control of it… [The MC trial] was more or less explained to me that…. I’d get a better quality of life because I’d be happier, and I wouldn’t be this and I wouldn’t be that.

(P03/02, M‐78, MC2‐Blinded)

Such expectations of ‘quality of life’ set the scene for patients’ situating of wellbeing and coping relative to MC, intersecting with desire—expressed through hope—for a returned sense of purpose and relational identity.

#### Hope and desiring bodies

Several interviewees described a balance of hope and realism in considering MC’s expected benefits to wellbeing. P01/24 (M‐54, MC1‐CBD) said ‘I don’t have great expectations’ but ‘anything that helps me is a bonus’. P01/04 (F‐81, MC1‐CBD) acknowledged that she was ‘grasping at straws perhaps’ but nonetheless wanted ‘to try it to see if the diagnosis can be eased a bit. Make me feel better’. For others, expectation was tempered by the uncertainty of RCTs and hope for a miraculous discovery:

I think it can probably help the cancer.… you never know, how it's going to go. But it's worth a try… I'm hoping it keeps me nice and relaxed and it does its job on the cancer… maybe stops it growing or at least… give[s] me a better quality of life.… I think that it can do all that. I believe it can.

(P01/10, F‐69, MC1‐CBD)

Contemplating popular media representations of MC as a ‘wonder drug’ in various contexts, P03/01 acknowledged his perception of the role of hope and positive thinking in quality of life and the potential for MC to impact this:

[Y]ou’re picking the positives to run because there’s not a lot of positives to run in cancer stories… so you pray for the drug trials.… I think if you weren’t positive about it then… you could well have a very short lifespan.… [Outlook] makes a big difference… to probably how long you live for, and also… [to] the quality of life that you have.

(P03/01, M‐57, MC1‐Placebo)

From ‘help’ with ‘feel[ing] better’ to a cure, hope connected to MC inflected the disconnection and anticipated re‐connection of the body‐relation‐emotion assemblage disrupted by cancer. MC‐hope acted as a potentially re‐stabilising force.

#### ‘Getting through their day’: Desiring bodies and normalcy

Emotional stability and regaining a sense of relational purpose and social identity underpinned other patients’ expectations of trial participation and MC’s effects on cancer symptoms. P01/17 (M‐58, MC1‐CBD), for instance, noted anticipatory pain as well as anxiety relating to his experience of cancer. When discussing his expectations of the trial medication he noted:

I’m quite happy to [participate in the trial]… because I do get quite [a bit of] anxiety and I think it’s partly to do with me, the kids and everything else that’s going on in life.

P01/17 (M‐58, MC1‐CBD)

Hope for a returned normalcy and a reconnected body‐relations‐emotion assemblage also inflected interviews with those who declined to participate in a MC RCT:

If there’s anything that can help someone get through the bad medical times, then I’m all for it… I’ve known some sick people and [they] have smoked [cannabis]… It helped with their pain and best of all, they were able to control it… If nothing else, I think that it would help people relax and help ease their pain and just help them get through their…days.

(P02/11, M‐66, Not on trial)

While not all interviewees saw MC as having the potential to improve their symptom burden and wellbeing, conceptualisations of wellbeing remained consistent with others’ descriptions of a state encompassing interconnected physical‐functional‐emotional‐relational desires:

Cannabis is going to have me off with the fairies… I’m not going to have a full life if I’m off in the clouds… [Wellness] is being able to live the life as I want… I find [when I play]… I’m thinking about golf and trying to hit a… shot, I’m not thinking about all the rest of the garbage going on….. That’s very important to my quality of life, and the social aspects of seeing mates and banter…. Quality of life… it’s just being able to live normal…. If I want to go the shops… I want to be able to do that. Not have to ring friends.

(P02/13, M‐70, Non‐trial)

Here, the point of divergence comes from contemplating the perceived role of MC in supporting a reclaimed ‘normal existence’—for this patient, MC represented a barrier to ‘being able to live normal’, in contrast to patients who saw it as potentially facilitating that returned normalcy.

Overall, although some patients expressed hope in MC’s anti‐cancer effects, they recognised that this was not the intent of the drug or the trial. In referencing the potential for MC to alleviate symptoms, act on the cancer (however improbably), and its more general effects on health and quality of life, however, patients again transcended—or transgressed—the targeted efficacy‐and‐safety assessment objectives and measures of the RCTs. Rather, they incorporated broader notions of wellness, coping, relational affect and mind‐body holism. This reflects an *assembling* of wellbeing through what Coffey (2022, p. 70) describes as ‘a process of engagements and relations, rather than [it] being held as a bodily property or possession’.

Further, patients situated themselves as desiring‐bodies in a Deleuzo–Guattarian sense. Desire ‘makes assemblages coalesce together’ but is also a ‘destabilising force that takes an assemblage apart’ (Muller & Schurr, 2016, p. 8), prompting the movement and repositioning of bodies. In this sense, patients acknowledged hopefulness relative to MC, as a means of orienting themselves relationally and emotionally to normalcy: Towards the experience and timescape of the living (Olson, Smith, Good, et al., 2022).

### ‘You plateau out’: Embodiment, empowerment, and experiences of medical cannabis

Both trial participants and non‐participants, who described using cannabis for therapeutic benefit, spoke of experiencing symptom relief from MC.

#### ‘Your body just winds down’: Medicinal cannabis on the trial

Patients participating in an RCT foregrounded mind‐body health, focusing on an overall sense of ‘wellbeing’ that encompassed MC’s reported or perceived effects on mental health, pain, discomfort, energy levels, coping and rational thought:

The cannabis oil relaxes me and I have a good night’s sleep… the painkillers do that but sometimes I wake up whereas being on the oil… I’m relaxed.… I’m not as moody and [family] has noticed that… I’m a bit… calmer, settled.

(P01/26, F‐52, MC2‐Blinded)

Participants articulated connections between physical relaxation, pain relief, a sense of coping, normality and wellbeing:

You sort of plateau out, mellow out, so you see past the issues and just deal with life as the moment… you get to a point where it doesn’t matter what’s going on up above you, below you. It’s where you are, and what you’re doing. That’s where I’m at, at the moment. …–[I]t’s not the illness that gets you, it’s all the other things around it that catch up with you…. Maybe if [patients] have access to medicinal marijuana, that part of their life could be better for them… It’s all about mind, body, and soul, treating those three things.

(Patient 01/18, M‐61, MC1‐CBD)

I feel relaxed. You know when your body just winds down? …that’s how I felt when I took it. I’d think, oh I like this… it is nice to relax… When I’m relaxed, my body’s relaxed… I had no pain… I feel normal.… it makes your life easier.

(P01/20, F‐55, MC1‐CBD)

Further, participants positioned MC relative to its potential contribution to emotional stability and lack of anxiety:

I’m a hell of a lot better in happiness… Anxiety, I couldn’t sleep at night because I only had this cancer thing on my brain. [MC] must be playing a part there… fixing that area up.… I’m quite happy with what’s happening to my body.…

(P03/02, M‐78, MC2‐Blinded)

Interviewees reflected on the nature of participation in an RCT while also providing a contrast through describing their experiences of MC within a holistic mind‐body wellness framework:

Whether I’m on a placebo or not I don’t care [if] my mind’s telling me it’s working.… I seem to think it’s working…. You get a better feeling of wellness… that it’s doing something… I just hope it’s not the placebo, but if it is well it’s just my brain saying it’s working. I don’t care.

(P01/07, M‐66, MC1‐CBD)

#### ‘It’s the sense of wellbeing that appeals’: Medicinal cannabis outside the trial

For those interviewees with non‐trial experience of using cannabis in a therapeutic setting (the vast majority of participants), there was a similar focus on easing anxiety, and occasional rumination on possible anti‐cancer effects: ‘I’ve had good results. Whether it’s from the cookies or from chemo, I don’t know.… I’ve found [MC] really good for anxiety at least… I think it’s excellent for that’ (P02/03, M‐65, Non‐trial MC).

Reduced worry and anxiety were also linked to an ability to think and act ‘more rationally’:

It doesn’t seem to have any effect except it stops you worrying. I used to have stress attacks.… I used to get very upset when people did something or got rude to me… I just don’t anymore. [MC]’s really made me much… more rational…. For a long time, I didn’t think it worked much… Then I realised it was actually knocking the pain out. Especially… background pain. Definitely making me feel much calmer… more rational… more patient.

(P02/12, F‐79, Non‐trial MC)

Building on MC’s predicted effects, in discussing the expected/experienced effects of MC on and/or off the trial, both RCT participants and non‐participants framed these effects in terms of embodied‐emotional‐rational functioning: relief from anxiety and improved sleep, relaxation and happiness. Thinking with Deleuzo–Guattarian theory here allows us to conceptualise MC as reassembling a feeling‐relating‐body disrupted by cancer; an entangled and situated (perceived) effect that defies numerical measurement.

## DISCUSSION

This sub‐study was designed to elicit patients’ reflections on MC and its use within and outside a clinical trial setting; however, as interviews and analysis progressed, intersecting perceptions and experiences of wellbeing became foregrounded. This paper describes an important point of articulation between bodies and assemblages, appreciating the hyper‐medicalised context of RCTs and related limitations in assessing ‘wellbeing’ and quality of life in that setting. Further, appreciation of this intersection fosters exploration of pain and other symptoms, and experiences of wellbeing and coping, with implications for how wellbeing is measured in RCTs. Finally, it affords a novel exploration of how patients conceptualise wellbeing through contemplation of the (re)assembling potential of MC—incorporating patients’ experiences and perceptions of MC in the clinical trial context and within a broader social milieu in which boundaries—legal‐illegal, acceptable‐unacceptable—of cannabis use are contested and shifting.

In discussing their experiences of cancer and its symptoms, patients described ‘pain’ as a kind of disassembling precipitated by interconnected losses: of physical function; emotional stability; social‐relational relevance; and of control over time, place and decision‐making. Pain and unwellness thus reflect an embodied subjectivity and ontological multiplicity; they are understood as diminishing bodily agency and an individual’s ability to choose their situatedness in terms of emotion, time, space and body‐function.

As a counterpoint to this loss and disassembling, interviewees’ conceptualisations of wellbeing highlighted a potential reassembling. Reclaimed wellbeing was conceived of as situated and future‐situating in emotions, spaces, the body and time. Further, the body was experienced as the site of affect and agency, hope and desire and resistance to the effects of illness and treatment (Grosz, 1995; Sointu, 2006). This embodiment of ontological multiplicity was parsed through patients’ references to pain as multi‐dimensional, prompting a critical reimagining of the biopsychosocial model and reorienting us to physical pain as also positional‐spatial‐relational. The physical and social are then always together, in and between bodies: subverting dualisms, foregrounding relational affect, and transgressing traditional notions of subjectivity, and particularly the subjectivity of pain and illness.

Patients’ contemplations of wellbeing are therefore not only a reflection of cancer’s impact on individual bodies. Rather, interviewees spoke repeatedly of loss and gain in ways that reflect a Deleuzo–Guattarian sense of bodies within the cartography of social domains: connecting and assembling, disconnecting and disassembling, reconnecting and reassembling through the processual flux of body practices. While a biopsychosocial model (Engel, [1977] 2012) helps consider the physical, psychological and interpersonal elements of cancer symptoms and their impact on wellbeing, it can do so in highly individualistic, pathologised, segmented and disembodied ways. As noted by Mcleod (2017), such an approach places responsibility for wellbeing—and blame for unwellness—with the individual. In the research presented here, by contrast, we employ the work of Deleuze and Guattari via Sointu (2016, 2006) and Coffey (2022).

Following Coffey (2022) in this study, wellbeing is conceptualised and experienced as not merely personal but in‐flux and inter‐corporeal: assembled and positioned relative to other bodies in the social field, constructed through reference to clinical and other discourses and dependent upon and always embodying relational affect. Patients actively positioned themselves relative to other physical‐social bodies and resituated their embodied and social sense of self. This (re‐)positioning was, further, temporal, with patients situating bodies in relation to their previous sense of self and their hopes for future relief and wellbeing. As such, a Deleuzo–Guattarian frame informs contemplation of bodies, wellbeing and of loss and gain in the context of advanced cancer and clinical trials, doing so through critical consideration of embodied, relational and reassembling e/affect.

Of particular, novel significance and patients’ reflections on MC in relation to wellbeing and cancer symptoms add contour to this social and affective cartography. Where cancer and associated symptoms are seen as generating loss and a *disassembling*, consideration of hoped‐for‐wellbeing and contemplation of MC’s potential is used as a means of parsing a reconceptualisation and engaging in a *reassembling*: A coalescing and repositioning underpinned by desire and (anticipation of) reclaimed agency. Shifting from conceptualisations of wellbeing more broadly, to an anchoring of wellbeing with specific reference to MC, is particularly illuminating because the broader frame of cannabis use—both recreational and medicinal—is characterised by the blurring of experiences and distinctions: legal‐illegal, medicinal‐recreational, acceptable‐unacceptable, prescribed‐obtained and medical‐alternative (Morris, 2020; Pedersen & Sandberg, 2013). Our work here suggests the usefulness of expansion beyond the predominant social science focus on stigma, decriminalisation and illicit drug use in the context of cannabis and MC, towards critical consideration of embodied MC assemblages.

At this point of articulation, cannabis use in highly medicalised contexts such as clinical trials might be more thoroughly and usefully interrogated as existing at a borderline. In this position, MC signals a back and forth movement between the personal, experiential, subjective and hoped‐for; and the clinical, measured and assessed. From a Deleuzo–Guattarian perspective (Buchanan, 1997; Deleuze & Guattari, 1987), those patients who contributed to this sub‐study can be seen as engaged in subverting dualisms and acknowledging and fostering multiplicity. Patients challenged not only mind/body/other conceptualisations as these might be encountered in the clinical assessment of the quality of life but also worked to reconceptualise time–space (Grosz, 1995) through reference to a multi‐dimensional assemblage comprised of current symptoms and anticipated relief and wellbeing. In this context, desire is critical: counterbalancing reflections on pain and loss and framing contemplations of wellbeing and the potential offered by MC. Of note, the intersecting and disruptive uncertainty and complexity around perception, expectation and experiences of MC were consistent across those who had taken part in an RCT and those who declined RCT participation. This suggests that MC itself is ‘useful to think with’ in the broader domain of research into experiences of advanced cancer.

This sub‐study responds to calls in social science scholarship to attend to experiential and ontological tensions apparent in patients’ experiences and understandings of cancer and cancer treatments. Moving beyond such calls, we employed a Deleuzo–Guattarian frame informed by Sointu (2013, 2006), Mcleod (2017) and Coffey (2022) to reconsider the sociological and clinical conceptualisation of wellbeing, with reference to patients’ experiences of accessing MC in a palliative cancer care and RCT setting. The above findings support the notion that experiences of cancer symptoms, and perceptions and experiences of MC, are complex, contested and highly contextualised. Our findings highlight a repeated focus by patients on a sense of loss of physical function and independence; social and relational purpose and roles; stability and predictability of emotions; and control over time, place and decisions. Patients’ perceptions and experiences of MC acted as a counterpoint to this loss–used by patients as a mechanism to parse both the meanings of these losses and the potential of trial participation to redress such loss.

There are acknowledged limitations in existing clinical approaches to understanding patient wellbeing, such as drawing on reductionist numerical pain scales that may conflate pain and function (Bhatia et al., 2014; Gibbins et al., 2013) or that segment ontologically intersecting experiences (Mescouto et al., 2022). Our findings point to the potential for bringing together recognition of the clinical realm with sociological approaches to patient perspectives on cancer and cancer symptoms. In particular, our research suggests a place for possible theoretical re‐sensitisation ahead of future MC RCTs and for new and different ways of appreciating patient perspectives and experiences in advanced cancer and palliative care. This lays a foundation for the future development of tools that might further capture the conceptual richness and experiential, embodied complexity of this domain from patients’ perspectives.

## CONCLUSION

In this paper, we advanced a Deleuz–Guattarian‐informed frame that enabled a critical focus on the intersecting embodied experiences of those who are affected by advanced cancer and its associated symptoms, including pain as a dominant experience. This frame enabled examination of wellness, wellbeing and quality of life, as they are situated within this setting. To achieve this, we considered several core components of Deleuze and Guattari’s work—including the concepts of complex assemblages and the operation of desire. Further, drawing on the writings of Grosz (1995), Sointu (2013) and Coffey (2022), we presented insights that highlight the significance of bodies and the situatedness of wellbeing, including patients’ conceptualisations of illness and wellness and, importantly, the role of MC relative to these. Here, wellbeing is situated as an affective–effective distillation of embodied subjectivity, present in the contemplations, understandings and experiences of those individuals considering their symptoms and the potential of relief through MC and a clinical trial. Our findings thus indicate the importance of shifting beyond conventional clinical scales often used within clinical trial settings to measure patients’ experiences and perceptions of pain and other symptoms, instead fostering consideration of the relation‐emotion‐body‐with‐cancer assemblage and accompanying articulations of wellbeing.

## AUTHOR CONTRIBUTIONS

**Alexandra Smith**: conceptualization (lead); formal analysis (lead); writing – original draft (lead). **Rebecca E. Olson**: conceptualization (supporting); formal analysis (supporting); funding acquisition (supporting); methodology (lead); supervision (lead); writing – original draft (supporting). **Nathalia Cordeiro da Costa**: conceptualization (supporting); writing – original draft (supporting). **Maddison Cuerton**: formal analysis (supporting); writing – review & editing (equal). **Janet Hardy**: funding acquisition (lead); writing – review & editing (equal). **Phillip Good**: funding acquisition (supporting); writing – review & editing (equal).

## CONFLICT OF INTEREST STATEMENT

The authors declare that they have no conflicts of interest.

## ETHICS STATEMENT

Ethical approval for this study was obtained from the Human Research Ethics Committees at the Mater Hospital (HREC/17/MHS/97) and St Vincent’s Hospital (HREC 17/27).

## PATIENT CONSENT STATEMENT

All participants provided their written consent.

## Data Availability

Data generated and analysed for the current study are available to suitably qualified individuals on request, from the corresponding author, subject to HREC approval.

## ENDNOTES

^1^
Acknowledging their status within the hospital, people who contributed to interviews are referred to as ‘patients’ or ‘interviewees’. ‘Participant’ and ‘non‐participant’ is reserved for references to interviewees’ RCT status.

^2^
Interviewee characteristics are represented using pseudonyms (P01/22), followed by gender, age (e.g., M‐57) and, if they participated in an RCT, the name of the trial (e.g., Pilot, MC1 or MC2) and intervention arm (e.g., placebo or CBD).
